# Field evaluation of the safety and compatibility of a combined vaccine against porcine parvovirus 1 and porcine reproductive and respiratory syndrome virus in breeding animals

**DOI:** 10.1186/s40813-019-0138-5

**Published:** 2019-12-16

**Authors:** Beatriz Garcia-Morante, Marta Noguera, Christian Kraft, Philip Bridger

**Affiliations:** 1 Centcinc Coworking, C/Montserrat de Casanovas 105, 08032 Barcelona, Spain; 2 Boehringer Ingelheim Veterinary Research Center GmbH & Co. KG, Bemeroder Straβe 31, 30559 Hannover, Germany

**Keywords:** Combined vaccine, Porcine parvovirus 1, Porcine reproductive and respiratory syndrome virus, Reproductive failure, Vaccine safety, Vaccine compliance

## Abstract

**Background:**

Porcine reproductive and respiratory syndrome virus (PRRSV) and porcine parvovirus 1 (PPV1) are two common causes of reproductive failure. ReproCyc® ParvoFLEX is a novel subunit vaccine based on the protective viral protein (VP) 2 of PPV1 that has been recently licensed in the European (EU) market, whereas ReproCyc® PRRS EU is a porcine reproductive and respiratory syndrome (PRRS) modified live virus (MLV) vaccine authorized in 2015. The present work sought to evaluate the safety and compatibility of the combined administration of the abovementioned vaccines in target animals under the context of a field PRRSV (experiment A) and PPV1 (experiment B) infection. To achieve this objective, safety and lack of vaccines’ antigen interference were established according to the absence of significant differences between the combined vaccinated animals (PPRSV+PPV1) and the single vaccinated animals against PRRSV or PPV1. In both experiments, gilts and sows were evaluated for local and systemic reactions after vaccination as well as for reproductive and productive performance. In addition, tissues from abortions, mummified fetuses and stillborn piglets were analyzed for the presence of PRRSV and PPV1. Lastly, serology and viremia were determined in experiment B.

**Results:**

No relevant differences in terms of safety, reproductive and productive performance between the single vaccinated and the combined vaccinated animals in either experiment were observed.

**Conclusions:**

ReproCyc® PRRS EU mixed with ReproCyc® ParvoFLEX can be used as a safe method of protection against the detrimental effects of PRRSV and PPV1 infections in breeding female pigs in one single injection. The present results also open up opportunities to tackle reproductive problems as a whole by combining control programs against swine reproductive pathogens.

## Background

Reproductive failure impairs the health and welfare of breeding pigs and impacts farmers’ gains worldwide. Viral infections are clearly the most common infectious cause of reproductive wastage, and porcine reproductive and respiratory syndrome virus (PRRSV), porcine circovirus type 2 (PCV2) and porcine parvovirus 1 (PPV1) infections are the most frequent ones [1]. Importantly, since vaccines are a valuable tool for prevention and decrease of the detrimental effects of the respective associated diseases [2–4], combined control strategies may be of interest for the swine production enterprise. Particularly, vaccinations against PRRSV and PPV1 are usually part of the control programs implemented in gilts and sows, thus, a combined strategy against these two pathogens is of special importance in breeding herds.

PRRSV is widely distributed among pigs and has been associated with resorption, late-term abortions, partially or completely mummified fetuses, stillborn and weak piglets at farrowing [5]. ReproCyc® PRRS EU (Boehringer Ingelheim Vetmedica GmbH, Ingelheim am Rhein, Germany) is a vaccine formulated from the porcine reproductive and respiratory syndrome (PRRS) type I modified live virus (MLV) strain 94881. This PRRS MLV vaccine has been already proven to be a safe and effective method of protection against the negative effects of PRRSV infection when administered to breeding female pigs [6, 7]. PPV1 is a ubiquitous and resistant virus with a wide range distribution, too. Its reproductive effects are generally not detected in immune adult animals. Hence, reproductive failure mainly occurs if infection is contracted during the first half of gestation in naïve females, especially in their first pregnancy, leading to mummified fetuses and resorptions. Infection after 70 days of gestation can lead to weak, infected piglets [2]. All vaccines available and used in Europe for preventing PPV1-induced reproductive failure consist of inactivated virus and they are generally considered safe and efficacious [8]. These vaccines, however, are based on PPV1 strains isolated several decades ago and they are adjuvanted with either oil or aluminum hydroxide [9, 10]. ReproCyc® ParvoFLEX (Boehringer Ingelheim Vetmedica GmbH, Ingelheim am Rhein, Germany) is a recently licensed monovalent subunit vaccine for pigs based on a recombinant baculoviral expression system producing the protective viral protein (VP) 2 of PPV1.

Both ReproCyc® PRRS EU and ReproCyc® ParvoFLEX vaccines are adjuvanted with carbopol (ImpranFLEX®^;^ BI Animal Health USA Inc., St. Joseph, MO, USA), a lightly cross-linked polymer of acrylic acid that has been shown to elicit robust humoral immunity and T-cell responses to some different types of veterinary vaccines [11–13]. The fact that both vaccines share the adjuvant supports their mixability, though the in-use stability and effectiveness of this mixture needs to be assessed. With the aim of developing a combination vaccine against PRRSV and PPV1 infections, we investigate in the present report the safety as well as putative interference of a combination comprising of both PRRS MLV and VP2 of PPV1 antigens when administered intramuscularly (i.m.) in the same injection. In this context, two vaccine field studies were performed in breeding gilts and sows in both a PRRSV (experiment A) and PPV1 (experiment B) field infection scenario. The comparison between results obtained from the combined vaccinated (PRRSV+PPV1) and the single-vaccinated animals against PRRSV or PPV1 served as a basis for safety and vaccines’ interference assessment. The herein presented results proved that vaccination of bred gilts and sows with ReproCyc® PRRS EU combined with the novel PPV1 subunit vaccine (ReproCyc® ParvoFLEX) is a safe option for preventing reproductive losses associated with PRRSV and PPV1 infections without compromising compliance of either vaccine.

## Materials and methods

### Studies design

#### Field experiment A: vaccine safety and compliance against PRRSV

The study was performed on a commercial farrow-to-finish farm in Spain as a positive-controlled, randomized and blinded trial in compliance with good clinical practice (GCP). The farm had a history of PRRSV circulation and outbreaks despite regular vaccination of the sows with a PRRS MLV vaccine. Two months before the study started, sera was obtained from 150 sows of different parity and tested for serology and viremia. Results confirmed seropositivity in 94.7% and PRRSV Type 1 viremia in 11.3% of the sampled sows. Sequencing of the gene open reading frame 5 (ORF 5) revealed the presence of two distinct PRRSV strains; one matching the vaccine strain and the other one considered a wild PRRSV Type 1. Besides, vaccination against PPV1 and *Erysipelothrix rhusiopathiae* was included in the routine vaccination scheme of lactating sows. On the study day (SD) 0, the whole breeding herd (not vaccinated against PRRSV within the last 3 to 4 months) was randomly assigned to two treatment groups (Table 1). A simple randomization at vaccination was performed as follows: animals were vaccinated in alternated order with ReproCyc® PRRS EU mixed with the PPV1 subunit vaccine (group A-PRRSV+PPV1; *n* = 297) or only with the PRRS MLV vaccine (group A-PRRSV; n = 297) and irrespective to their reproductive stage. Every tenth animal per treatment group, one was allocated as a “sample animal”. Consequently, 10% (*n* = 30) of the sows and gilts from each group were randomly assigned to a safety evaluation group designated as “sample animals”. The animals were monitored until the end of the study, i.e. up to 4 months from vaccination (SD 122).

**Table 1:** Field experiment A and B design. In experiment B, the study was conducted in two phases, in which the enrolled animals were split into two treatment groups across phase I or phase II

		Group	N	Study day (SD)			
Experiment A				0	7	21	90
		A-PRRSV+PPV1	297	Mixed vaccine^a^	–	–	–
		A-PRRSV	297	PRRS MLV vaccine^b^	–	–	–
Experiment B	Phase I	B_I_-PRRSV+PPV1	118	Mixed vaccine	Placebo^c^	PPV1 subunit vaccine	PRRS MLV vaccine
		B_I_-PPV1	120	PPV1 subunit vaccine^d^	PRRS MLV vaccine	PPV1 subunit vaccine	PRRS MLV vaccine
	Phase II			201	208	298	
		B_II_-PRRSV+PPV1	138	Mixed vaccine	Placebo	PRRS MLV vaccine	
		B_II_-PPV1	145	PPV1 subunit vaccine	PRRS MLV vaccine	PRRS MLV vaccine	

^a^ReproCyc® PRRS EU combined with ReproCyc® ParvoFLEX

^b^ReproCyc® PRRS EU

^c^Sodium chloride solution (NaCl; FisioVet®, B Braun VetCare, S.A., Rubí, Spain)

^d^ReproCyc® ParvoFLEX

#### Field experiment B: vaccine safety and compliance against PPV1

The study was performed on three commercial farms located all in Spain. It was designed as a parallel positive-controlled, randomized and blinded trial in compliance with GCP. All farms had a history of routine vaccination against PPV1 once a year, 7 to 10 days after farrowing. The source for the gilts was internal replacement in all three farms. Gilts from two farms came from different nucleus herds from the same company while the third one produced its own gilt replacement stock. In order to monitor for PPV1 circulation in all three enrolled sites, blood samples of at least 10 non-vaccinated replacement stock gilts were periodically obtained during the study and tested by ELISA and PCR. The proportion of seropositive gilts varied between sampling time points in all farms, from 0 to up to 100%, which confirmed the existence of unprotected gilt subpopulations susceptible to PPV1. Importantly, seropositive gilts older than 6 months and viremic gilts were present in all three sites at some point. Since screened gilts were not vaccinated, it was assumed that positives titers after the age of 6 months reflected a field infection on farm as maternal antibodies are supposed to have disappeared by this time [14].

The study was conducted in two phases in which the enrolled animals were split into two treatment groups across phase I or phase II (Table 1). All included gilts in phase I were not previously vaccinated against PPV1 and PRRSV. On the contrary, all included sows in phase II were previously vaccinated against PPV1 and PRRSV according to immunization schedules at each individual farm. Because of ethical and economic considerations, the PPV1 mono-vaccinated groups were also vaccinated against PRRSV (SD 7). In phase I (*n* = 238), the primary vaccination (i.e. prime and boost vaccination 3 weeks apart) with ReproCyc® ParvoFLEX alone (group B_I_-PPV1) or in combination with ReproCyc® PRRS EU (group B_I_- PRRSV+PPV1) was applied to gilts (at least 6 weeks prior mating). The insemination of these gilts was done according to normal husbandry practices of the farm and they were monitored up to weaning of their offspring. Phase II (*n* = 283) comprised re-vaccination with the PPV1 subunit vaccine alone (group B_II_-PPV1) or mixed with ReproCyc® PRRS EU (group B_II_- PRRSV+PPV1) of a subset of sows primo-vaccinated in phase I (*n* = 55) together with newly enrolled multiparous sows (*n* = 228) vaccinated in the previous gestation with a different commercial vaccine against PPV1. Again, sows were followed up until weaning of their offspring. Furthermore, some gilts and sows from each group and phase were randomly selected as “sample animals” for evaluation of safety parameters; 62 (26%) and 81 (34%) animals were assessed in phase I and II, respectively.

### Vaccination

In both field experiments, the licensed PRRS MLV vaccine (ReproCyc® PRRS EU) from commercial batches was used. Conversely, the PPV1 subunit vaccine (ReproCyc® ParvoFLEX) was produced for experimental purposes only. For the combination of both vaccines, the freeze-dried cake of ReproCyc® PRRS EU was reconstituted with the PPV1 subunit vaccine, resulting in a liquid suspension containing the same amount of ImpranFLEX® than the original solvent for ReproCyc® PRRS EU. All treatments were administered i.m. into the neck musculature caudal of the ear base and at a volume of administration of 2 ml per animal. As blinded studies, all injections were performed by a dedicated administrator not involved with data collection.

### Criteria for safety assessment

In both field experiments, safety was evaluated by means of monitoring clinical signs, rectal temperatures and local reactions at the injection sites principally in “sample animals”. The frequency of the observed parameters was compared between treatment groups.

#### Clinical observations of general health

In both experimental studies, clinical signs of disease were assessed as formerly described [6], including “behavior”, “respiration”, “digestion” and “other” as categories. In experiment A, clinical observations of all the animals were recorded from SD 0 to 14, and then weekly throughout the study. On SD 0 (vaccination day), clinical observations were done before vaccination and 4 h after. In experiment B, clinical observations were recorded from “sample animals”; in phase I, from SD 0 to 14, and SD 21 to 35 and in phase II, from SD 201 to 215 (i.e. 2 weeks after single or combined PPV1 vaccination). On SD 0, 21 and 201 (single or combined PPV1 vaccination days), clinical observations were done before and 4 h after vaccination.

#### Rectal temperatures

Temperatures were measured rectally in “sample animals” using self-calibrated digital thermometers at approximately the same time each day and recorded in degrees Celsius (°C) units. In both experiments, rectal temperatures were obtained before the start of the study or phase, and the calculated mean temperature was used as a covariate (baseline) for post-treatment time points. Thus, pyrexia was defined as an increase in temperature of ≥1.5 °C respective to the baseline temperature. In experiment A, rectal temperatures were taken before (SD 0) and 4 h after (SD 0 + 4 h) vaccination. After treatment, rectal temperatures were measured daily for a week (SD 1 to 7). Similarly, in experiment B, rectal temperatures were obtained at PPV1 (single or in combination) vaccination days (SD 0, 21 and 201), before and 4 h after injection. Following the aforesaid time points, rectal temperatures were measured daily for a week (SD 1 to 7, 22 to 28 and 202 to 208).

#### Injection site observations

All injection sites were examined in “sample animals” for redness, swelling, and secretion. The approximate size of the injection site reaction was also recorded using a conventional ruler. Injection site inspection was performed at the same time points than clinical signs assessment but for experiment A, injection sites were observed from SD 0 to 14 but not weekly throughout the study.

### Conception and abortion rate

Artificial insemination of all gilts and sows was recorded at an individual level. Those gilts and sows that did not conceive or were suffering from abortion during the period of implantation (around 14 to 35 days post-service) were recorded as “returned to service”. Repeated inseminations in single animals between 35- and 120-days post-service were recorded as an “abortion”. The mean conception and abortion rates per group were calculated and compared.

### Farrowing performance

In both field experimental studies, farrowing data were recorded for each gilt and sow. Day of farrowing for each animal was defined as the day that the first piglet was delivered. At the time of farrowing, each piglet was classified into one of five categories listed below: “mummy”, “stillborn”, “crushed”, “weak live”, “healthy live”. The proportion of piglets in each category at farrowing was compared between treatment groups.

### Weaning rate

The number of piglets alive at weaning was recorded for each gilt and sow. If possible, cross-fostering was limited to sows within the same treatment group. Anyhow, cross-fostered piglets were marked and were re-allocated to the mother sow at weaning for precise recording. The mean proportion of piglets per litter at weaning was calculated per treatment group to allow comparisons between them.

### Serology and viremia assays

Blood was collected by jugular venipuncture from “sample animals” in experiment B. Blood samplings were performed before the start of the study (SD − 1), before SD 21 (2nd day of PPV1 vaccination) and at farrowing in phase I, whereas in phase II, animals were sampled before SD 201 (1st day of vaccination) and at farrowing. Also, blood was monthly collected along both phases. Blood samples were processed for serum and tested for PPV1-VP2 antibodies using a commercially available blocking enzyme-linked immunosorbent assay (bELISA; INgezim® PPV1 Compac, INGENASA, S. A, Madrid, Spain) according to the manufacturer’s recommendations. Samples were also tested for PRRSV by quantitative real-time polymerase chain reaction (qPCR; bioScreen GmbH, Münster, Germany) as previously reported [6, 15] and for PPV1 by means of a conventional polymerase chain (PCR) reaction method described earlier [28].

### *Postmortem* tissue collection and viral detection

In both field studies, lung and/or kidney tissues were analyzed for the presence of PRRSV by qPCR (experiment A) or for PPV1 by conventional PCR (experiment B) in those litters with more than two mummified fetuses and/or stillborn piglets. Moreover, when an abortion occurred, tissue samples of the placenta and aborted fetuses were obtained to be also investigated for PRRSV and PPV1. Frozen tissue samples were stored in appropriate containers, labelled and tested by the same PRRSV-qPCR and PPV1-PCR methods than those performed on sera.

### Statistical analysis

The statistical analyses and data summaries were done using SAS software, version 8.2 (SAS Institute, Cary, NC, USA). All data for both experiments were summarized descriptively (*N* = sample number, mean, standard deviation [STD] or confidence interval [CI]) based on the type of variable. All data were analyzed assuming a completely random design structure and tests on differences were designed as two-sided tests at α = 5%, with differences considered significant if *P* ≤ 0.05.

For statistical purposes, each individual gilt or sow was used as the experimental unit in both experiments, though the litter was the experimental unit for reproductive performance evaluation. The main objective of experiment A was to compare the combined vaccinated group A-PRRSV+PPV1 with the mono-vaccinated group A-PRRSV in a PRRSV field infection scenario. Likewise, experiment B focused on the comparison of combined vaccinated animals (groups B_I_ and B_II_-PRRSV+PPV1) with the single vaccinated animals (groups B_I_ and B_II_-PPV1) in each phase and under a PPV1 field infection scenario. Specifically, generated frequency tables for clinical observations, pyrexia, injection site reactions, return to service, abortions and weaned piglets were analyzed using Fisher’s exact test, while mean rectal temperatures and differences in reproductive performance between groups were tested by the analysis of covariance (ANCOVA) derived t-test and the Wilcoxon-Mann-Whitney test, respectively. Additionally, PPV1 bELISA results from experiment B were tested by means of Fisher’s exact test.

## Results

In both experiments, there were animals removed mainly due to site economic reasons and as a part of the routine breeding farms’ management. Data of animals that were removed from either trial were included in their respective parameter of analysis until removal. In experiment A, a total of 98 sows were excluded from the study (48 sows from group A-PRRSV+PPV1 and 50 sows from group A-PRRSV). None of the removals belonged to “sample animals”. In experiment B, a total of 92 gilts and/or sows were removed from the study; 51 during phase I and 41 during phase II. In phase I, one of the removals was a “sample animals” from group B_I_-PPV1.

### Safety parameters

#### Clinical observations of general health

Most of the clinical signs recorded from “sample animals” and both studies belonged to the category “other”, which for instance included poor body condition, hernia and lameness. Indeed, lameness was the most frequent clinical sign recorded in both field experiments. Importantly, no statistical difference between treatment groups regarding clinical observations were found in experiment A nor in experiment B. The proportion of clinical observations per category and treatment group in each experiment is available as Additional file 1.

#### Rectal temperatures

In experiment A, the mean rectal temperature of “sample animals” ranged between 38.3 and 38.5 °C in both groups, hence, not showing statistical differences between treatment groups. Only 4 animals evenly distributed over both groups had pyrexia starting 4 h after injection and not lasting longer than two consecutive days. Elevated temperatures resolved without any treatment in all cases.

**Fig. 1:**
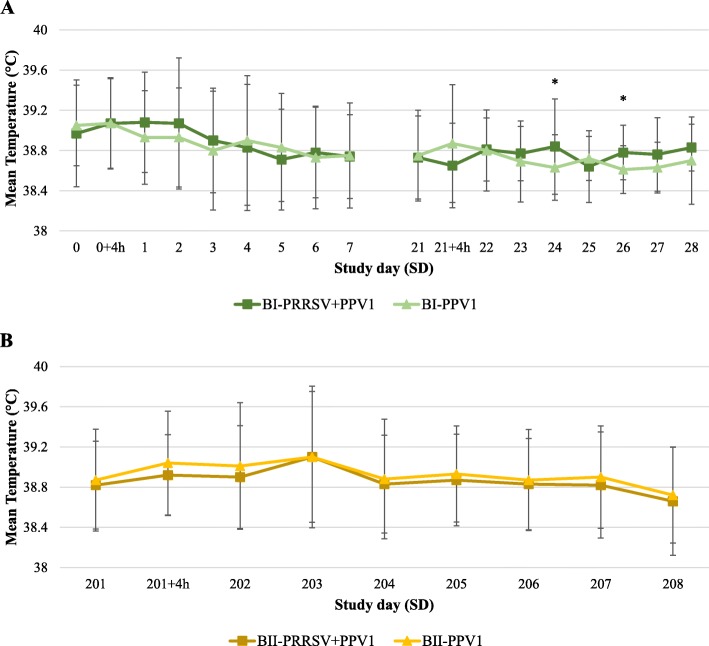
Mean (±STD) rectal temperatures of “sample animals” per group, from phase I (**a**) and phase II (**b**) of experiment 2. *Statistically significant differences (*P* ≤ 0.05) by ANCOVA derived t-tests

Mean rectal temperatures of “sample animals” within each group from experiment B are shown in Fig. 1a and b for phase I and II, respectively. In phase I, comparisons between groups revealed significantly higher temperatures of group B_I_-PRRSV+PPV1 on SD 24 (*P* = 0.034) and 26 (*P* = 0.011). In contrast, no significant differences between treatment groups were found along phase II. In total seven animals had a rectal temperature increase of 1.5 °C or higher: four from phase I and three from phase II. After PPV1 1st vaccination (SD 0), temperature elevation was observed in two and one animals from groups B_I_-PPV1 and B_I_-PRRSV+PPV1, respectively. After 2nd vaccination (SD 21), only one animal belonging to the B_I_-PPV1 group showed a rectal temperature increase of at least 1.5 °C. After re-vaccination in phase II (SD 201), 2 animals from the B_II_-PRRSV+PPV1 group and 1 from the B_II_-PPV1 showed pyrexia. Remarkably, in all cases, the rectal temperature increase did not last longer than two consecutive days and it was not linked to any other abnormal clinical sign or injection site reaction. Increased temperatures resolved without any treatment.

#### Injection site reactions

From experiment A, redness or secretion was not recorded at any time from either group. A total of three animals all belonging to the group A-PRRSV showed slight swellings (never above 2.5 cm of diameter) at the injection site from one to a maximum of four consecutive days. Nonetheless, no statistical differences between treatment groups were observed in terms of swelling frequency.

**Fig. 2:**
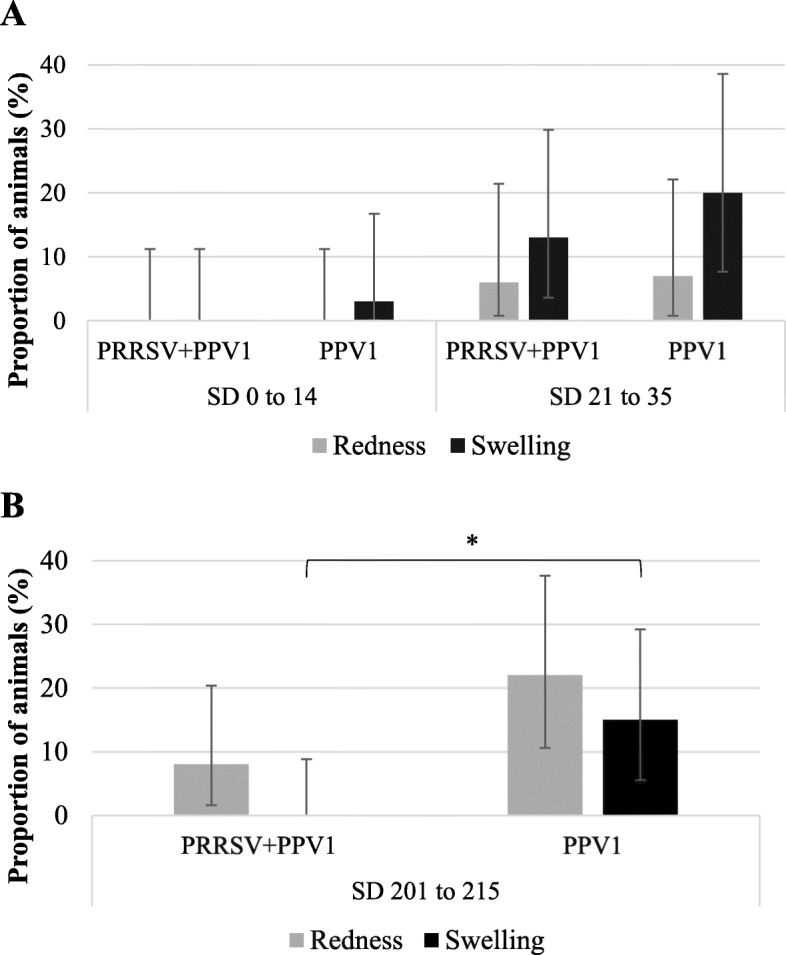
Frequency (±CI 95%) of injection site reactions in “sample animals” per group, from phase I (**a**) and phase II (**b**) of experiment B. *Statistically significant differences (*P* ≤ 0.05) by Fisher’s exact test

Frequency of injection site reactions from experiment B by treatment group are shown in Fig. 2a and b for phase I and II, respectively. Secretion was not recorded at any time from either group and redness and/or swelling did not last more than 3 days in any animal. While no statistical differences were seen between groups in phase I, the frequency of animals with swelling at the injection site was significantly lower (*P* = 0.026) in group B_II_-PRRSV+PPV1 when compared with group B_II_-PPV1 in phase II.

### Conception and abortion rate

Conception and abortion rate results from both field experiments are summarized in Table 2. Proportion of gilts and sows returned to service within 35 days after insemination was not statistically different between groups in experiment A nor in experiment B. Likewise, proportion of gilts and sows that suffered abortions (returned to service after 35 days from insemination) was not statistically different between groups in any of the field studies.

**Table 2:** Reproductive performance**.** Proportion (%) of return to service (≤ 35 days) and abortions (≥ 35 days) of gilts and sows from experiments A and B (phase I and II)

			Return to service					Abortions				
	Group	N_total_	N_returned_	%_returned_	95% CI^a^		*P*^*b*^	N_abortions_	%_abortions_	95% CI		*P*^*b*^
Experiment A	A-PRRSV+PPV1	269	19	7.1	4.3	10.8	0.628	3	1.1	0.2	3.2	1.000
	A-PPV1	265	22	8.3	5.3	12.3		2	0.8	0.1	2.7	
Experiment B phase I	B_I_-PRRSV+PPV1	108	8	7.4	3.3	14.1	0.408	3	2.8	0.6	7.9	0.683
	B_I_-PPV1	109	5	4.6	1.5	10.4		2	1.8	0.2	6.5	
Experiment B phase II	B_II_-PRRSV+PPV1	136	4	2.9	0.8	7.4	1.000	2	1.5	0.2	5.2	0.498
	B_II_-PPV1	130	4	3.1	0.8	7.7		0	0.0	0.0	2.8	

^a^Confidence interval

^b^*P*-value of Fisher’s exact test for comparison between groups

### Farrowing and weaning performance

**Fig. 3:**
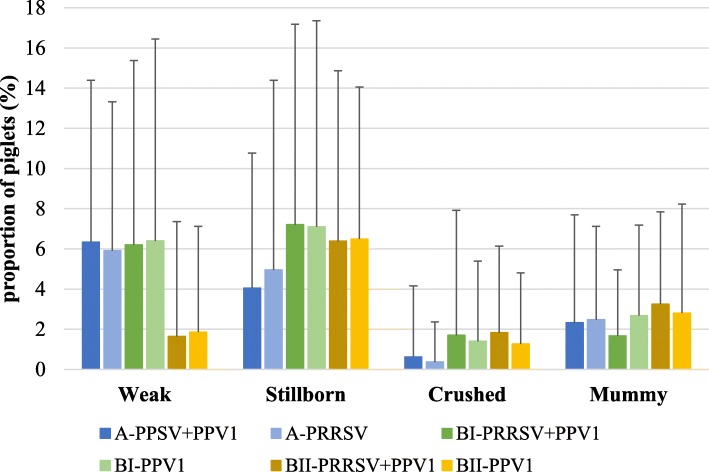
Mean (±STD) proportion of piglets per litter in each abnormal farrowing performance category from experiment A and B (phase I and II). The remaining percentage of piglets per litter correspond to piglets born and classified as “healthy”. *P*-values of Wilcoxon Mann-Whitney test for comparison between groups reveled no statistically significant differences at any time point

The mean absolute number of piglets alive per sow at farrowing and at weaning in each treatment group from both field studies is shown in Table 3. Differences were not statistically significant between treatment groups in any of the two experiments. Besides, the mean proportion of piglets per litter in each abnormal farrowing performance category from experiment A and B is depicted in Fig. 3. The proportion of piglets in each category was not statistically different between treatment groups in any of the two trials.

**Table 3:** Farrowing and weaning performance. Absolute numbers of alive piglets at farrowing and at weaning per sow from experiments A and B (phase I and II)

		Farrowing					Weaning				
	Group	N_total_	Mean	95% CI^a^		*P*^b^	N_total_	Mean	95% CI^a^		*P*^b^
Experiment A	A-PRRSV+PPV1	249	13.4	13.0	13.8	0.567	249	11.0	10.6	11.5	0.898
	A-PPV1	247	13.5	13.1	13.9		247	11.1	10.7	11.5	
Experiment B phase I	B_I_-PRRSV+PPV1	92	14.7	14.0	15.4	0.466	90	12.6	11.8	13.3	0.470
	B_I_-PPV1	99	15.2	14.5	15.9		97	12.6	11.9	13.4	
Experiment B phase II	B_II_-PRRSV+PPV1	125	14.5	13.8	15.1	0.095	124	12.0	12.3	86.1	0.416
	B_II_-PPV1	120	15.2	14.5	15.8		118	12.5	13.2	85.2	

^a^Confidence interval

^b^*P*-value of Wilcoxon Mann-Whitney test for comparison between group

### Serology and viremia

**Fig. 4:**
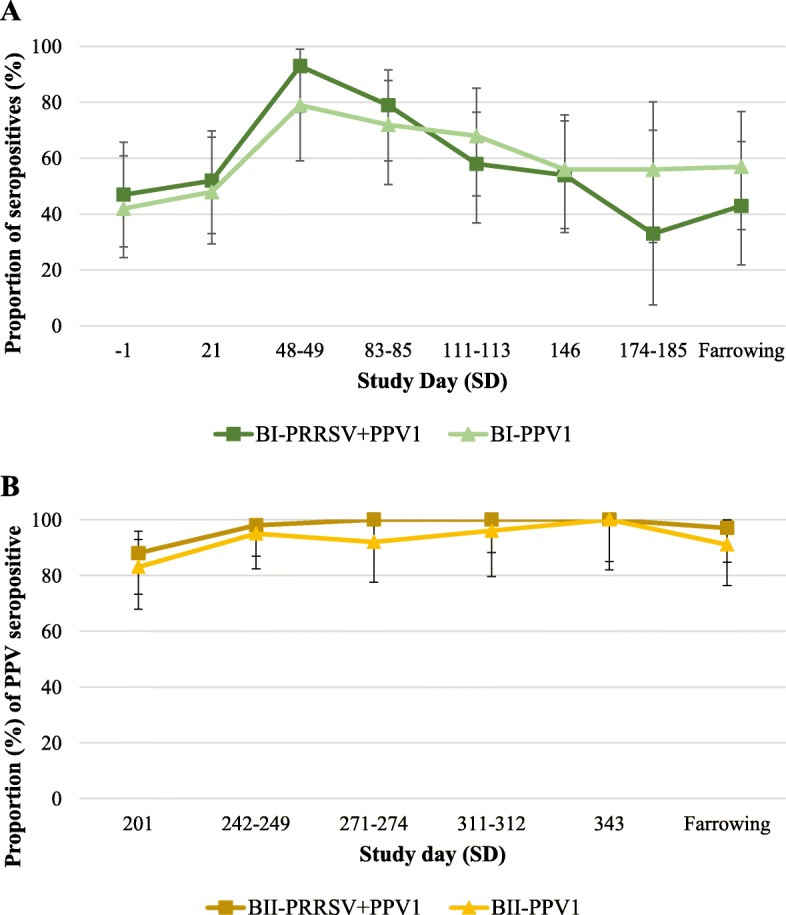
Proportion (±CI 95%) of seropositive animals against PPV1 per group, from phase I (**a**) and phase II (**b**) of experiment B. In phase I (**a**), primary vaccination (SD 0) with ReproCyc® ParvoFLEX alone (group B_I_-PPV1) or in combination with ReproCyc® PRRS EU (group B_I_- PRRSV+PPV1) and boost vaccination with ReproCyc® ParvoFLEX alone 3 weeks apart (SD 21). In phase II (**b**), re-vaccination (SD 201) with ReproCyc® ParvoFLEX alone (group B_II_-PPV1) or mixed with ReproCyc® PRRS EU (group B_II_- PRRSV+PPV1). Values of Fisher’s exact test for comparison between groups reveled no statistically significant differences at any time point

Proportion of seropositive animals against PPV1 from experiment B are depicted in Fig. 4a and b for phase I and II, respectively. No statistical differences were noted between groups in phase I nor in phase II at any time point. Furthermore, none of the blood samples analyzed were positive to PPV1 or PRRSV by PCR or qPCR.

### Virus detection in tissue samples

In study A, there were 14 sows from group A-PRRSV+PPV1 and 23 from group A-PRRSV that had more than two mummies and/or stillborn piglets at farrowing, thus, tissue samples were collected and analyzed accordingly. All but two lung samples tested negative for PRRSV by qPCR. These positive samples derived from two mummified fetuses of the same sow belonging to the group A-PRRSV. In addition, five sows had an abortion (see Table 2) and all samples collected from their fetuses were negative to PRRSV and PPV1.

In phase I of study B, there were 15 gilts and/or sows in group B_I_-PRRSV+PPV1 and 13 in group B_I_-PPV1 with the abovementioned characteristics at farrowing and consequently sampled. Furthermore, tissue samples from mummies and stillborn piglets were obtained in phase II from 15 and 13 sows in groups B_II_-PRRSV+PPV1 and B_II_-PPV1, respectively. All collected tissue samples tested negative for PPV1 by PCR. A total of five (phase I) and two (phase II) sows aborted during the trial (see Table 2) and all tissue samples taken from their fetuses were PPV1 and PRRSV negative by PCR and qPCR, respectively.

## Discussion

Successful swine production aims to deliver as many liveborn piglets as possible by, in part, preventing infectious diseases affecting reproductive performance. Among the most relevant are those caused by viruses and recognized principally by their effect on pregnant gilts and sows. In this sense, PRRSV and PPV1 are two of the dominating pathogens in the global industry of breeding pigs [1, 2]. Further, PRRSV and PPV1 are capable of enhancing the virulence of PCV2 [16–19] and one or both are often present on porcine circovirus-associated disease affected farms [20, 21]. Thus, vaccines against PPRSV and PPV1 are used extensively in breeding herds and farmers may routinely vaccinate sows (pre-farrow or post-farrow) or may vaccinate the entire herd at regular intervals (mass vaccination). In such a context, the combined application of vaccines is considered a substantial advantage regarding animal welfare and human resources as well as in the economization of the production costs.

Reprocyc® ParvoFLEX is a recently licensed monovalent subunit vaccine for pigs based on a recombinant baculoviral expression system producing the protective VP2 protein of PPV1. This novel vaccine is adjuvanted with carbopol (ImpranFLEX®), which indeed is found in most of FLEX Family™ vaccines and other vaccines such as ReproCyc® PRRS EU. The fact of sharing a component would theoretically make the mixability between such vaccines easier. Nevertheless, interaction between vaccines against swine pathogens has been described earlier [22] and combinations must be safe, and its compliance not compromised. Subsequently, the aim of the present study was to test a combined vaccination against PRRSV and PPV1 to evaluate safety and compatibility of different vaccination schemes in the event of a PRRSV (experiment A) and a PPV1 (experiment B) field infection scenario.

Primary criteria for evaluating safety included local and/or systemic reactions to vaccination whereas putative interaction between vaccines was determined by evaluating reproductive performance at farrowing and at weaning. This was supported by evaluating gilts and sows for PPV1 serology and viremia in experiment B as well as by testing *postmortem* tissues from abortions, mummified fetuses and/or stillborn piglets for PRRSV and/or PPV1 in both experiments. Safety and vaccines’ compliance were established according to the lack of significant differences between the combined vaccinated animals and the mono-vaccinated animals. According to these criteria, the present data demonstrated that vaccination of bred gilts and sows with ReproCyc® PRRS EU combined with the novel PPV1 subunit vaccine (ReproCyc® ParvoFLEX) is a safe option for preventing reproductive losses associated with the PRRSV and the PPV1 infections.

The safety and effectiveness of the EU type PRRS MLV vaccine in gilts and/or sows that were in either early or late pregnancy has been already evaluated in trials that involved a challenge of PRRSV [6, 23] or a field natural infection [24–26]. One of the most important factors for obtaining registration for the combined use of a MLV PRRS vaccine is to ensure that the PRRSV is kept alive after the vaccines are mixed together ensuring the mixture in-use stability. Henceforth, field trial A was conceived to assess the combined safety and compliance against PRRSV of ReproCyc® PRRS EU when applied mixed with the PPV1 subunit vaccine. Combined PRRSV and PPV1 vaccinated animals from experiment A exhibited neither increased incidence of local nor systemic reactions after vaccination when compared to their single PRRSV vaccinated counterparts, revealing that the administration of the mixture is safe. Similarly, no differences were devised in terms of conception and abortion rates, farrowing performance and number of weaned piglets between treatment groups. Thus, no signs of interference between vaccines were observed which suggest viability of the PRRSV after mixing ReproCyc® PRRS EU and ReproCyc® ParvoFLEX.

Even though the vast majority of organ tissues from abortions, mummies and/or stillborn piglets at farrowing were PRRSV negative, two positive lung samples derived from two mummy piglets of a single PRRSV vaccinated sow were found in study A. The positive tissue samples were subjected to sequencing efforts; unfortunately, the sequencing reactions were unsuccessful as the amount of genetic material was likely not sufficient to generate a PCR product to be sequenced. Therefore, it could not be discerned if a natural PRRSV infection took place as it has been described that vaccine type 1 PRRS MLV may replicate in the host causing viremia in breeding females, which can lead to transplacental infection of fetuses [6, 23]. It is worth mentioning, however, that in the farm where experiment A took place, vaccination against PRRSV was not in place for piglets, thus, the unvaccinated fattening pigs might have provided a source of unprotected animals for virus circulation.

The inactivated PPV1 vaccines currently licensed are based on NADL-2 and similar strains, and were isolated 40 years ago [9, 27]. These vaccines are effective against homologous infections, but do not prevent infection and virus shedding after challenge with antigenically heterologous strains [9]; vector, subunit or MLV vaccines might be alternative approaches. ReproCyc® ParvoFLEX has been recently tested to be safe and efficacious under experimental conditions [28]. Nevertheless, its safety as well as in-use stability when applied mixed with ReproCyc® PRRS EU in the context of a PPV1 field infection has not been demonstrated so far.

Regarding safety assessed in experiment B, significantly higher rectal temperatures in the combined vaccinated group were found at two time points (i.e. SD 24 and SD 26) through phase I compared to rectal temperatures in the mono-vaccinated group. Due to the minor numerical differences as well as to the inherent variability in the measured variable, the aforesaid statistical differences were considered not biologically relevant. Hence, the administration of the PPV1 subunit vaccine alone or associated with the PRRS MLV vaccine in gilts and sows resulted in no clinically relevant differences in systemic reactions. Concerning injection site reactions, the separate but consecutive administration of ReproCyc® ParvoFLEX (SD 201) and ReproCyc® PRRS EU (SD 208) in phase II led to a significantly higher frequency of animals with swelling when compared to the animals vaccinated with the mixture once. The later support the combined vaccination as a tool for reducing putative injection site reactions, labor and stress of injecting pigs multiple times. In terms of PPV1 subunit vaccine’s compliance, no differences were observed in conception and abortion rates, farrowing performance and number of weaned piglets when different groups were compared. Importantly, all blood samples as well as all tissue samples collected from aborted, mummy and/or stillborn piglets at farrowing were negative for PPV1. Tissue samples from abortions were also PRRSV negative. Thus, while PPV1 or PPRSV as cause of abortion could be excluded, abortion could result from interplay of endocrine systems and dam-fetus pathophysiology or could be influenced by other infectious or noninfectious insults [4].

If gilts develop active immunity by PPV1 natural or vaccine exposure prior to conception, PPV1 would seldom be a problem. However, if they are first exposed to PPV1 anytime during about the first half of gestation, transplacental infection and reproductive failure are likely to arise [2]. While hardly anything is known about the role of cellular immunity, the effective clearance of PPV1 infection is known to be obtained by rapid antibody response in infected pigs [9, 27, 29, 30]. In experiment B, between 40 and 50% of the gilts in phase I were seropositive to PPV1 before the start of the study, revealing exposure to the pathogen. The proportion of PPV1-seropositive pigs barely increased after primo-vaccination (SD 0), though seropositive gilts increased steadily after the PPV1 boost (SD 21) in both vaccinated groups. Furthermore, serological results from phase II show that a bi-annual PPV1 re-vaccination (alone or in combination with PRRSV) of already immunized animals may help to reach 100% of protection. Anyhow, seronegative gilts from phase I did not show clinical signs associated with PPV1 and no viremia was observed, thus, suggesting protection beyond humoral immunity. All results together highlight the in-use stability of ReproCyc® ParvoFLEX when mixed with ReproCyc® PRRS EU.

The occurrence of the concurrent natural infections involving PRRSV and PPV1 in pigs has been described [1, 31] and it has been recently suggested that coinfection of PPV1 with PCV2 or PRRSV could occur resulting in more severe reproductive failure and neonatal mortality [32]. Thus, there is a potential for use of a combination vaccine for control of these infections. Under this scope, the present studies provide new insights in the combined fight against PRRSV and PPV1. Despite the present results cannot be sidelined, it is important to bear in mind that vaccine outcomes might be affected by several factors (e.g. infection pressure on the farm, virus circulating strains, co-infection with other swine pathogens, management practices and housing conditions). Therefore, control and prevention programs should be tailored to each individual farm and extrapolation of results should be done very carefully.

## Conclusion

Combination vaccines are an effective means of decreasing the number of injections and simplifying the immunization schedule, thus providing overall benefits concerning animal welfare, labor and costs. The present results point out that the combined vaccination of ReproCyc® PRRS EU with the innovative PPV1 subunit vaccine (commercially available as ReproCyc® ParvoFLEX) applied to gilts and multi-parity sows resulted in no inferiority for any of the safety nor compliance parameters evaluated when compared to the same vaccines applied separately. Henceforth, any negative interference between vaccines could be subtracted. These new data should interest EU swine producers in their global combat against reproductive failure in domestic pigs.

## Supplementary information

**Additional file 1: **Proportion (%) of animals with clinical observations within each category and the overall within each group from experiment A and B (phases I and II). *P*-values of Fisher’s exact test for comparison between groups reveled no statistically significant differences.

## Data Availability

The data that support the findings of this study are available from Boehringer Ingelheim Animal Health, Inc. but restrictions apply to the availability of these data, which were used under license for the current study, and so are not publicly available. Data are however available from the authors upon reasonable request and with permission of Boehringer Ingelheim Animal Health, Inc.
