# Supplementary material for: Field evaluation of the safety and compatibility of a combined vaccine against porcine parvovirus 1 and porcine reproductive and respiratory syndrome virus in breeding animals
Source: Porcine Health Manag. 2019 Dec 16;5:28. doi: 10.1186/s40813-019-0138-5 (PMC6916098; doi:10.1186/s40813-019-0138-5)
Supplement: Supplementary file 1 — Additional file 1: Proportion (%) of animals with clinical observations within each category and the overall within each group from experiment A and B (phases I and II). P-values of Fisher’s exact test for comparison between groups reveled no statistically significant differences. [file 40813_2019_138_MOESM1_ESM.docx]

|  | **Group** | **Behavior** | **Respiration** | **Digestion** | **Other** | **Total** |
| --- | --- | --- | --- | --- | --- | --- |
| **Study A** | A-PRRSV+PPV1 | 0.0 | 0.0 | 1.7 | 5.4 | 5.7 |
|  | A-PPV1 | 0.0 | 0.3 | 1.3 | 6.1 | 6.7 |
| **Study B**  phase I  (SD 0 to 14) | B_I_-PRRSV+PPV1 | 0.0 | 0.0 | 0.0 | 3.2 | 3.2 |
|  | B_I_-PPV1 | 0.0 | 0.0 | 0.0 | 12.9 | 12.9 |
| **Study B**  phase I  (SD 21 to 35) | B_I_-PRRSV+PPV1 | 3.2 | 3.2 | 0.0 | 16.1 | 16.1 |
|  | B_I_-PPV1 | 0.0 | 0.0 | 0.0 | 10.0 | 10.0 |
| **Study B**  phase II  (SD 201 to 215) | B_II_-PRRSV+PPV1 | 0.0 | 0.0 | 27.5 | 35.0 | 42.5 |
|  | B_II_-PPV1 | 0.0 | 0.0 | 22.0 | 39.0 | 51.2 |

**Additional file 1**. Proportion (%) of animals with clinical observations within each category and the overall within each group from experiment A and B (phases I and II). *P*-values of Fisher's exact test for comparison between groups reveled no statistically significant differences.
